# Spatiotemporal variation in vegetation phenology and its response to climate change in marshes of Sanjiang Plain, China

**DOI:** 10.1002/ece3.9755

**Published:** 2023-01-18

**Authors:** Yiwen Liu, Xiangjin Shen, Jiaqi Zhang, Yanji Wang, Liyuan Wu, Rong Ma, Xianguo Lu, Ming Jiang

**Affiliations:** ^1^ Northeast Institute of Geography and Agroecology Chinese Academy of Sciences Changchun China; ^2^ University of Chinese Academy of Sciences Beijing China

**Keywords:** climatic change, marsh, phenology, response, Sanjiang Plain, vegetation

## Abstract

Sanjiang Plain is the largest marsh distribution area of China, and marshes in this region significantly affect regional carbon cycle and biodiversity protection. The vegetation phenology of marsh significantly affects the energy exchange and carbon cycle in that region. Under the influence of global climatic change, identifying the changes in phenology and their responses to climatic variation in marshes of Sanjiang Plain is essential for predicting the carbon stocks of marsh ecosystem in that region. Using climate and NDVI data, this paper analyzed the spatiotemporal variations in the start (SOS), end (EOS), and length (LOS) of vegetation growing season and explored the impacts of climatic variation on vegetation phenology in marshes of Sanjiang Plain. Results showed that the SOS advanced by 0.30 days/a, and EOS delayed by 0.23 days/a, causing LOS to increase significantly (*p <* .05) by 0.53 days/a over marshes of Sanjiang Plain. Spatially, the large SOS advance and EOS delay resulted in an obvious increasing trend for LOS in northern Sanjiang Plain. The rise of spring and winter temperatures advanced the SOS and increased the LOS, and the rise in temperature in autumn delayed the EOS in marshes of Sanjiang Plain. Our findings highlight the necessity of considering seasonal climatic conditions in simulating marsh vegetation phenology and indicate that the different influences of climatic variation on marsh vegetation phenology in different regions should be fully considered to assess the marsh ecosystem response to climatic change in Sanjiang Plain.

## Graphical abstract

Under the influence of global climatic change, identifying the changes in phenology and their responses to climatic variation in the marsh of Sanjiang Plain is essential for assessing the carbon stocks of marsh ecosystems in that region. Using climate and NDVI data, this paper analyzed the spatiotemporal variations in the start (SOS), end (EOS), and length (LOS) of the vegetation growing season, and explored the responses of vegetation phenology to climatic variation in marshes of the Sanjiang Plain.

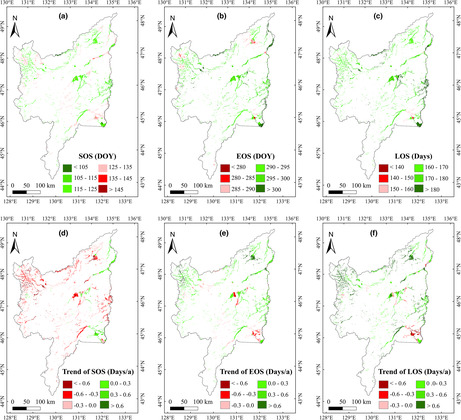

## 1 INTRODUCTION

Marsh is an important type of wetland ecosystems, which plays a key part in protecting biodiversity, regulating runoff, and changing global climate (Chatterjee et al., 2015; Erwin, 2009; Hu, Borsje, et al., 2021; Junk et al., 2013; Lewitus et al., 1998; Natuhara, 2013; Saderne et al., 2021; Sarkar & Borah, 2018; Smith & Kirwan, 2021). Vegetation is an important component of marsh ecosystem (Marani et al., 2013; Shen, Xue, et al., 2019; Shen et al., 2021; Zeng et al., 2022). Marsh vegetation phenology significantly affects energy exchange, ecosystem functions, and carbon cycling (Vázquez‐Lule & Vargas, 2021). Vegetation phenology of marsh is significantly susceptible to climatic variation, and it has altered significantly in recent decades due to climatic variation (Mo et al., 2015). Under the background of climatic variation, identifying the spatiotemporal variations of marsh phenology and understanding climatic effects on marsh phenology are essential for studying the regional carbon cycle (Luo, 2007; Shen, Liu, et al., 2019).

Sanjiang Plain is the largest marsh distribution area of China, and these marshes affect the regional carbon cycle and biodiversity protection (Luo et al., 2022). Global climate change has significantly altered the marsh in this region (Wang et al., 2011), which may significantly impact the regional carbon sequestration and ecosystem functions (Shen, Liu, et al., 2019). The effect of climatic variation on farmland vegetation phenology in Sanjiang Plain was analyzed, and it was observed that temperature promoted the advance of the vegetation growth period (Li et al., 2012). The effect of climate variation on forest phenology in Sanjiang Plain was studied, an increase in spring temperature advances the start of vegetation growing season (SOS), and an increase of autumn temperature delays the end of growing season (EOS) in Sanjiang Plain (Guo & Hu, 2022). Marsh ecosystems have unique climatic and environmental conditions compared with other ecosystems (Shen, Liu, et al., 2021), which may lead to different effects of climatic variation on vegetation phenology. The phenology in the marsh of Sanjiang Plain has been previously investigated. The SOS of *Carex lasiocarpa* was mainly concentrated in May (Sun & Song, 2008). The EOS of *Carex lasiocarpa* and *Deyeuxia angustifolia* were mainly concentrated in October (Hao et al., 2006). However, most researchers studied the plant phenology of few species in Sanjiang Plain, and no studies have investigated the vegetation phenological changes over marsh of Sanjiang Plain. Many researchers have indicated that climate change has different effects on the phenology of different vegetation types in one area or even on the same vegetation in diverse areas (Shen, Liu, et al., 2019). Until recently, spatiotemporal variations of marsh vegetation phenology and climatic effects in Sanjiang Plain have not been clearly identified yet. Therefore, it is necessary to study spatiotemporal changes in phenology and climatic effects in marshes of Sanjiang Plain.

In this work, we used NDVI and climate data (2001–2020) to analyze the spatiotemporal variations of marsh SOS, EOS, and length of growing season (LOS) in Sanjiang Plain. We also investigated the relationships between climate factors and vegetation phenology in Sanjiang Plain's marshes. The purpose of this study was to quantify the variations of vegetation phenology and explore the effects of climatic variation on phenology in Sanjiang Plain's marshes.

## 2 MATERIALS AND METHODS

### 2.1 Study area

**FIGURE 1:**
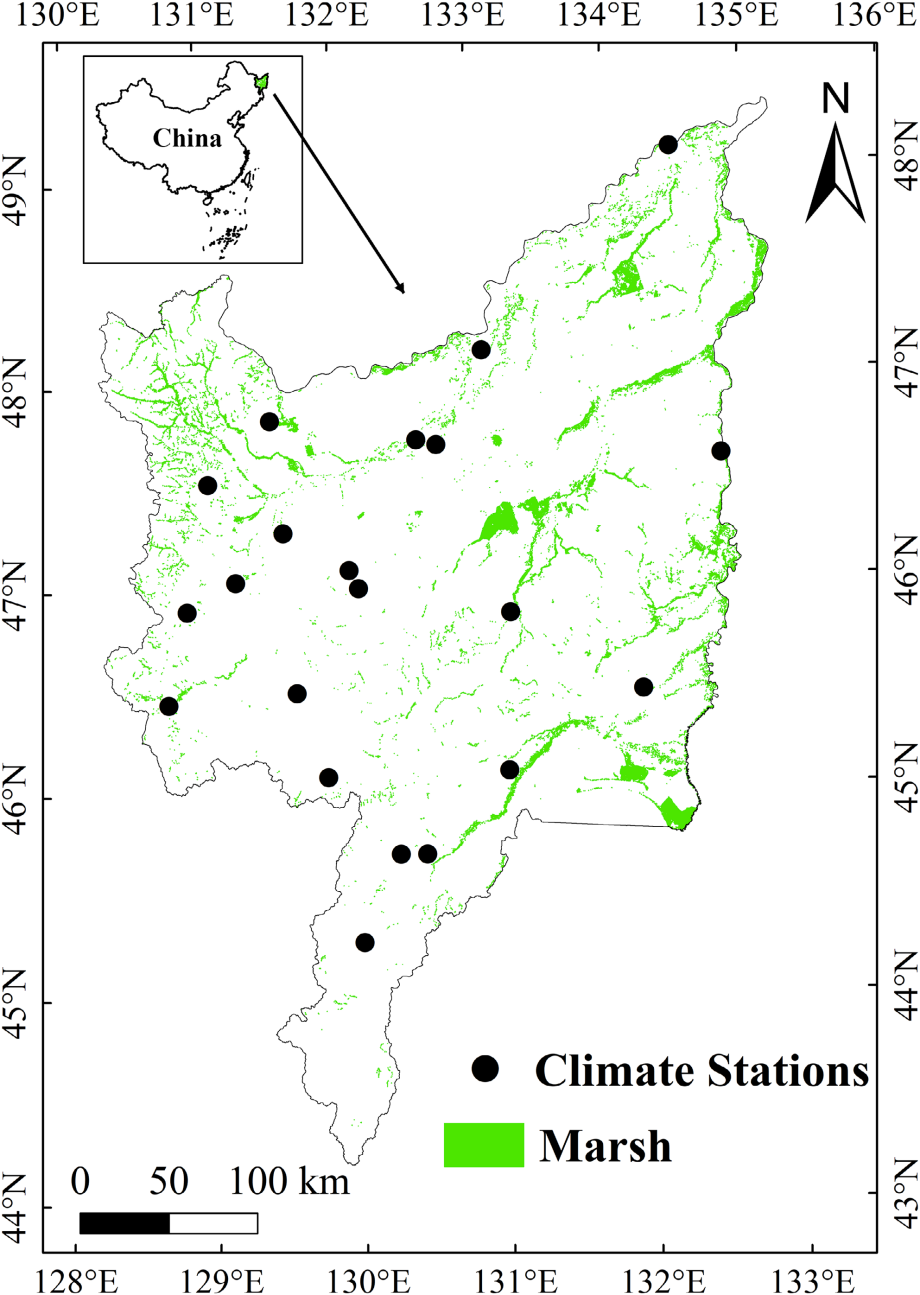
Distribution of marsh and climate stations in Sanjiang Plain of China

Sanjiang Plain is situated in northeastern China with longitude and latitude of 129°11′E–135°05′ E, 43°49′N–48°27′N (Figure 1). The climate in study area is continental monsoon climate, with annual mean temperature from 1 to 5°C, and approximately 60% of the precipitation is concentrated in the months of July and August (Hu, Zhang, et al., 2021; Wu et al., 2021). The common marsh species in Sanjiang Plain are *Carex lasiacarpa*, *Calamagrostis anagustifolia*, and *Carex meyeriana* (Song et al., 2014).

### 2.2 Data

#### 2.2.1 The climatic data

This research used monthly average temperature and precipitation data (2001–2020) from 21 climate stations in study region (Figure 1). The meteorological data are downloaded from the China Meteorological Administration (http://www.nmic.cn/en), and underwent strict quality assurance (Shen, Liu, et al., 2019).

#### 2.2.2 The MODIS NDVI data

This study extracted the phenology using the MOD13Q1 NDVI data (2001–2020), which have been widely used to extract vegetation phenology because of their good performance (Bhandari et al., 2011; Cao et al., 2018; Duan et al., 2018; Enkhzaya & Tateishi, 2010; Galvão et al., 2016; Hwang et al., 2014; Lebrini et al., 2019; Muche et al., 2019; Oliveira et al., 2016; Pringle et al., 2012; Sarvia et al., 2021; Shen et al., 2009). The NDVI data's temporal and spatial resolutions are 16 days and 250 m, respectively (Shen, Liu, Henderson, et al., 2022). To minimize errors and deviations, we performed initial quality control on these MODIS NDVI datasets based on pixel reliability parameters (Shen, Liu, et al., 2019).

#### 2.2.3 The distribution of freshwater marsh data

Two marsh maps in 2000 and 2015 covering Sanjiang Plain (Mao et al., 2020) were used in this study. The spatial resolution of marsh distribution data was 30 m, and the accuracy has been verified in combination with field survey data (Mao et al., 2020).

### 2.3 Methods

#### 2.3.1 The extraction of phenology

For the purpose of avoiding the influences of land use/cover variation on our results, we extracted unchanged marshes (pertaining to marsh in both 2000 and 2015) during the study period and chose them as study area. Consistent with previous researches (Piao et al., 2006, 2017; Shen et al., 2018; Shen, Liu, et al., 2019; Ma, Shen, et al., 2022; Su et al., 2022), we used the polyfit‐maximum approach to determine the phenology of marshes. First, we calculated the average of NDVI and analyzed the temporal variation in NDVI based on the formula (1):

(1) $$\text{NDVIrate}\left(t\right)=\frac{\text{NDVI}\left(t+1\right)-\text{NDVI}\left(t\right)}{\text{NDVI}\left(t\right)}$$

Here, t refers the Julian date (DOY), and NDVI (*t*) represents NDVI variation. When the NDVI rate reaches the largest increase/decrease date of the NDVI at this time is used as the threshold to determine average start and end date of growing season. We calculated the LOS using SOS and EOS of marsh vegetation in Sanjiang Plain.

As it is impacted by some nonvegetation effects of cloud, solar radiation angle, atmosphere and other factors, the NDVI detected by remote sensing usually has some outliers (Piao et al., 2006). We used the polynomial maximum method to better fit the NDVI time series. Its formula is as follows:

(2) $$\text{NDVI}=a_{0}+a_{1}d+a_{2}d^{2}+\ldots +a_{6}d^{6}$$

where a_1_,…a_6_ refer the fitting coefficients of the least square regression.

#### 2.3.2 The calculation of climatic data

We applied the ordinary Kriging approach to interpolate the climatic data of weather stations into the marsh distribution of Sanjiang Plain, and then unified the spatial resolution of interpolated climate dataset into the same as NDVI dataset (Liu et al., 2022). Monthly climatic data were used to calculate mean values of temperature and precipitation in winter (previous December–February), autumn (September–November), summer (June–August), and spring (March–May).

#### 2.3.3 The trend analysis

For each variable, we calculated the regional average value based on the mean of all pixels distributed in this study region. This study used linear regression to calculate the change trends in variables (2001–2020) as following (Shen, Liu, Zhang, et al., 2022; Shen, Xue, et al., 2019).

(3) $$\theta \text{slope}=\frac{\left(n\times \sum_{i=1}^{n}i\times \mathrm{Mi}\right)-\left(\sum_{i=1}^{n}i\times \sum_{i=1}^{n}\mathrm{Mi}\right)}{n\times \sum_{i=1}^{n}i^{2}-{\left(\sum_{i=1}^{n}i\right)}^{2}}$$

Here, *M*_*i*_ refers to the variables value in the ith year; *n* means the period length and is 20 in this work; *i* refers the serial number of year; *θ*_slope_ is the variation trend in variables of each pixel, if *θ*_slope_ > 0, it means that the variables have an increasing trend, otherwise it is reduced.

#### 2.3.4 The relationship between phenology and climate factors

This study used a Pearson's correlation analysis to explore the impacts of climate change on marsh phenology (Shen, Jiang, Lu, & Zhang, 2022).

(4) $$\mathrm{Rab}=\frac{\sum_{i=1}^{n}\left(\mathrm{ai}-\bar{a}\right)\left(\mathrm{bi}-\bar{b}\right)}{\sqrt{\sum_{i=1}^{n}{\left(\mathrm{ai}-\bar{a}\right)}^{2}}\sqrt{\sum_{i=1}^{n}{\left(\mathrm{bi}-\bar{b}\right)}^{2}}}$$

Here, *R*_*ab*_ is correlation coefficient; *n* refers to the period length and is 20, *a*_*i*_ represents the mean value of the climatic factors in year *i*; $\bar{a}$ means the average climatic factors during the past 20 years; *b*_*i*_ means the phenology in year *i*; $\bar{b}$ refers to the average phenology during the past 20 years.

## 3 RESULTS

### 3.1 Distribution of marsh phenology in Sanjiang Plain

**FIGURE 2:**
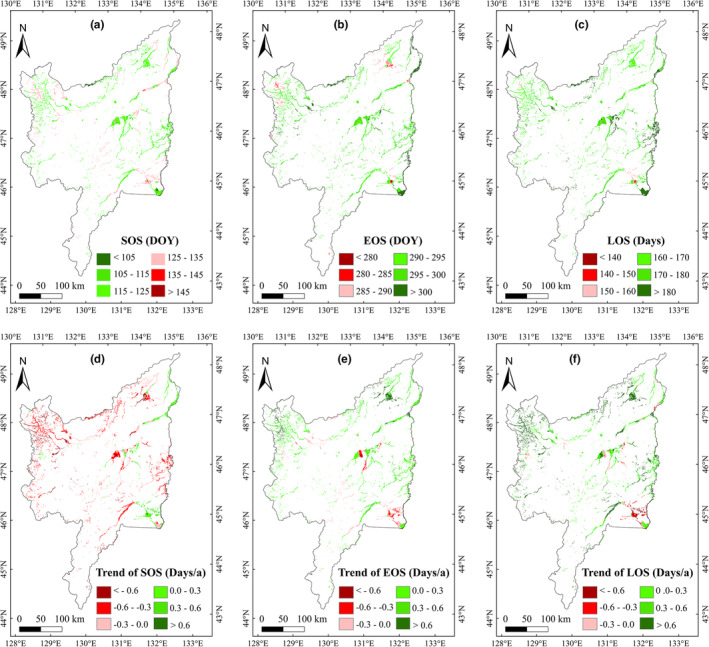
Spatial distributions of average (a–c) and temporal trends (d–f) of marsh vegetation phenology in Sanjiang Plain during 2001–2020

The average SOS and EOS of marsh vegetation in Sanjiang Plain were approximately 122 DOY (May 2 and 1 for nonleap and leap year, respectively) and 296 DOY (October 23 and 22 on nonleap and leap year, respectively), respectively, during 2001–2020. Thus, the average LOS of marsh vegetation was approximately 174 days over the study area. Spatially, SOS was mainly concentrated in 105–135 DOY, and the region with earlier SOS was mainly found in the east of study area (Figure 2a). By contrast, EOS was mainly concentrated in 285–305 DOY, and the region with later EOS was mainly found at eastern Sanjiang Plain (Figure 2b). Accordingly, LOS was mainly concentrated in 155–190 days, and the region with a long LOS was mainly concentrated in the east of study area (Figure 2c).

### 3.2 Trend in marsh vegetation phenology of Sanjiang Plain

**FIGURE 3:**
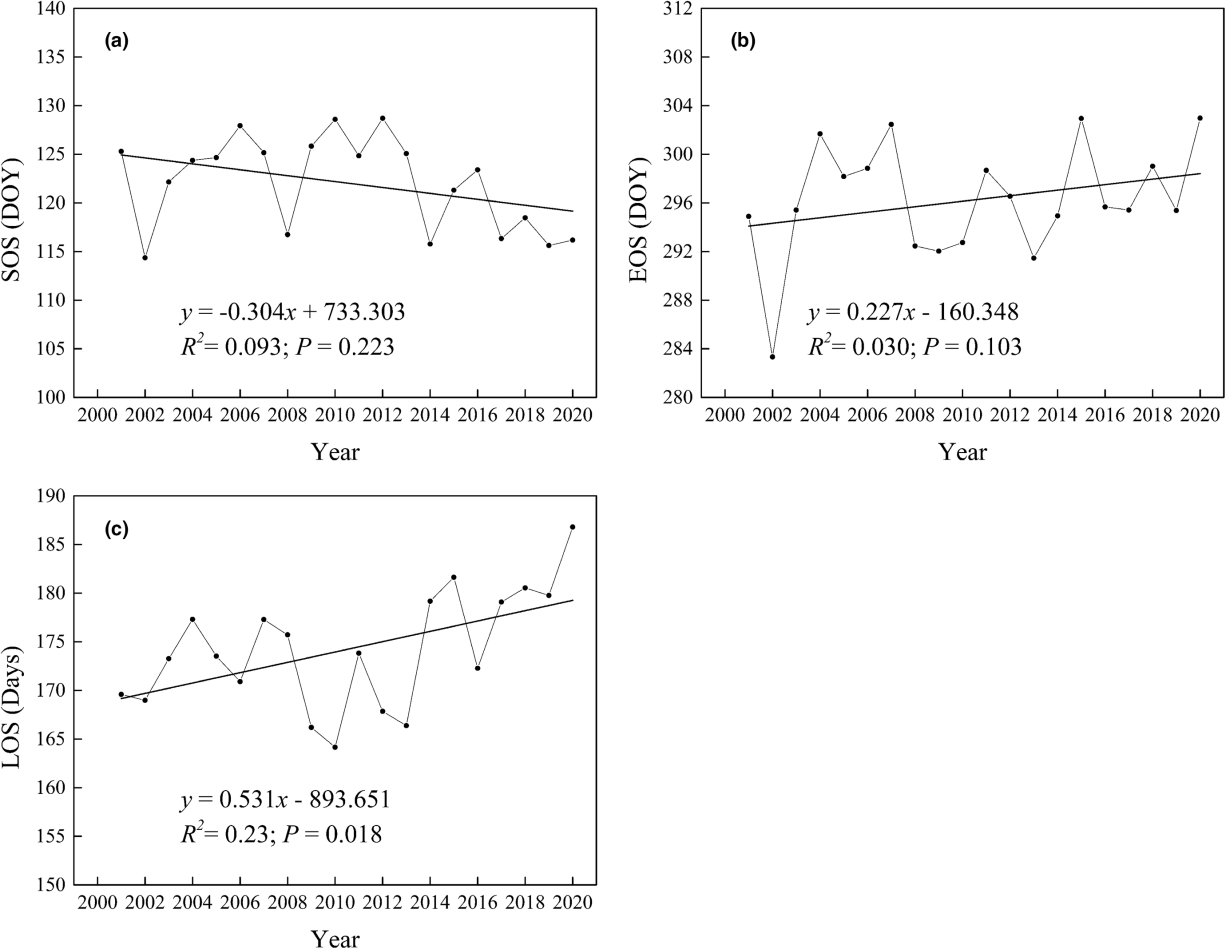
Temporal changes in vegetation phenology in marshes of Sanjiang Plain (2001–2020)

The marsh SOS advanced by 0.30 days/a (Figure 3a), and EOS delayed by 0.23 days/a (Figure 3b) in Sanjiang Plain (2001–2020). The LOS showed a significant (*p* < .05) increased by 0.53 days/a (Figure 3c). Spatially, the region with the largest advance trend of SOS was distributed in the north Sanjiang Plain. The region with the delayed trend was mainly located in the middle and southeast of Sanjiang Plain (Figure 2d). Furthermore, with regard to the changes in EOS, the region with the largest delayed trend of EOS was located in the north study area, and the region with the early trend was mainly located in the middle and southeast of study region (Figure 2e). The region with an increasing LOS trend was distributed in the north Sanjiang Plain. The region with a decreasing trend was located in the middle and southeast of study region (Figure 2f).

### 3.3 Correlations between meteorological elements and marsh vegetation phenology in Sanjiang Plain

**FIGURE 4:**
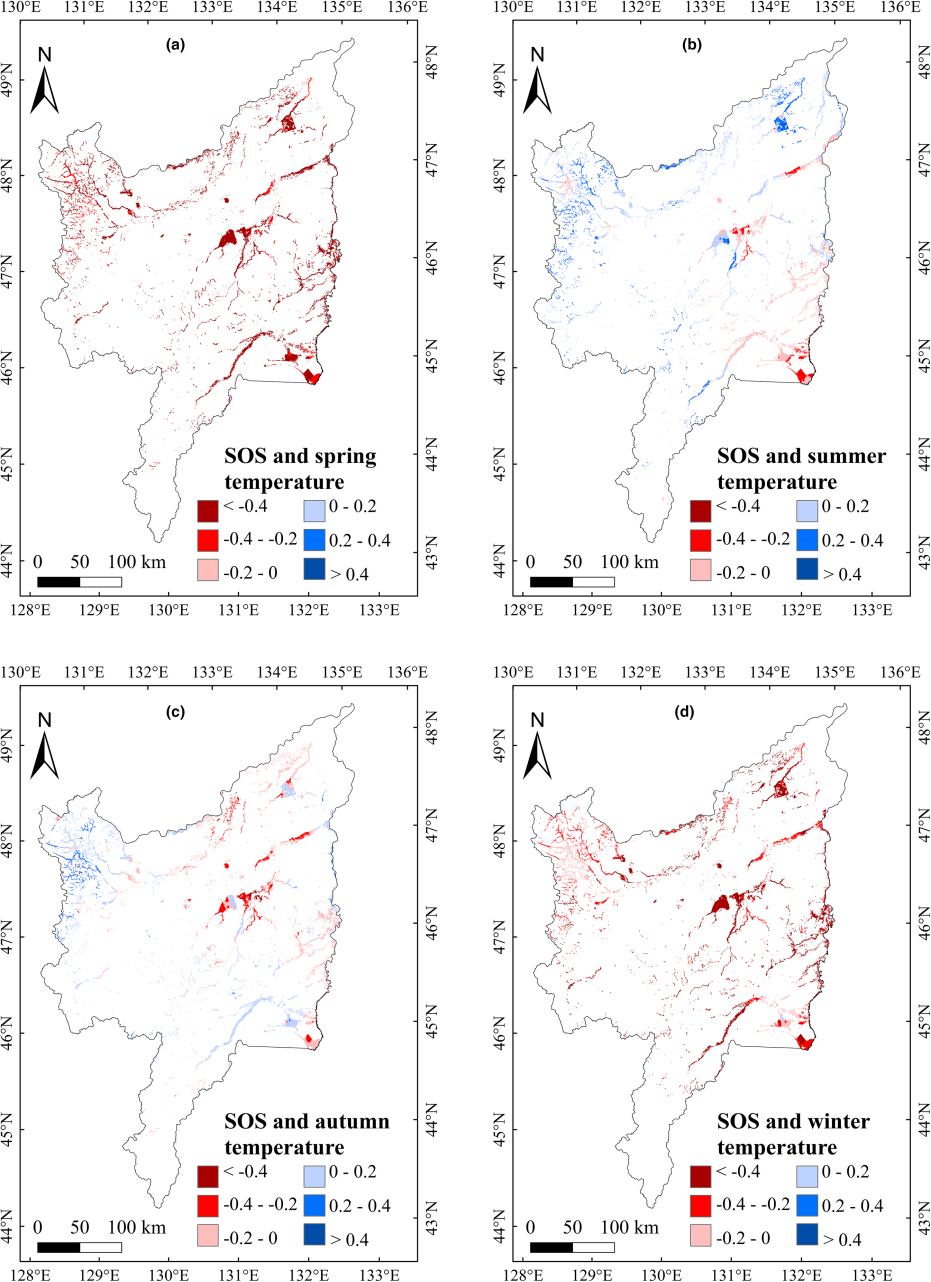
Correlations between SOS and seasonal average temperature in marshes of Sanjiang Plain (2001–2020)

**FIGURE 5:**
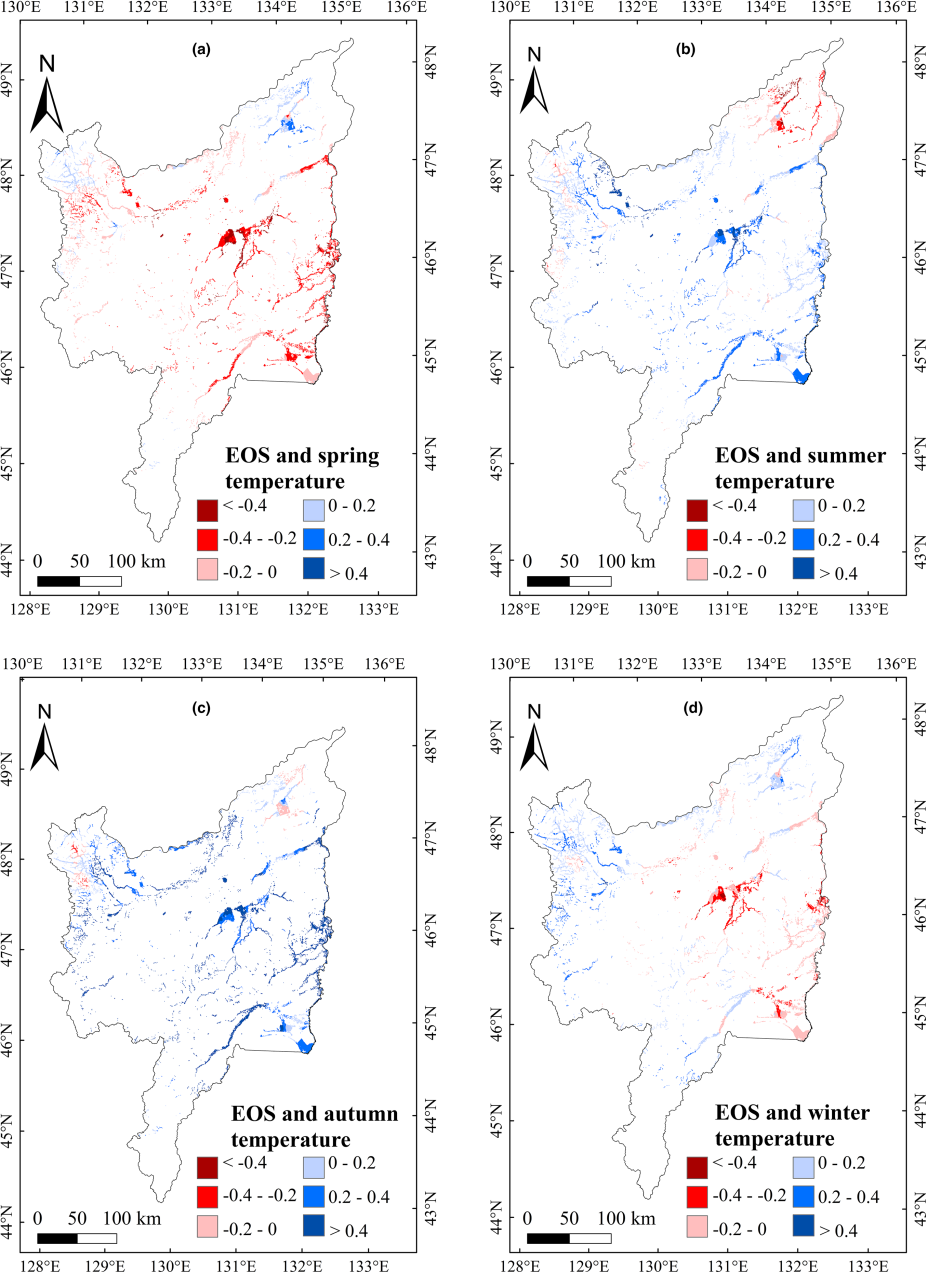
Correlations between EOS and seasonal average temperature in marshes of Sanjiang Plain (2001–2020)

**FIGURE 6:**
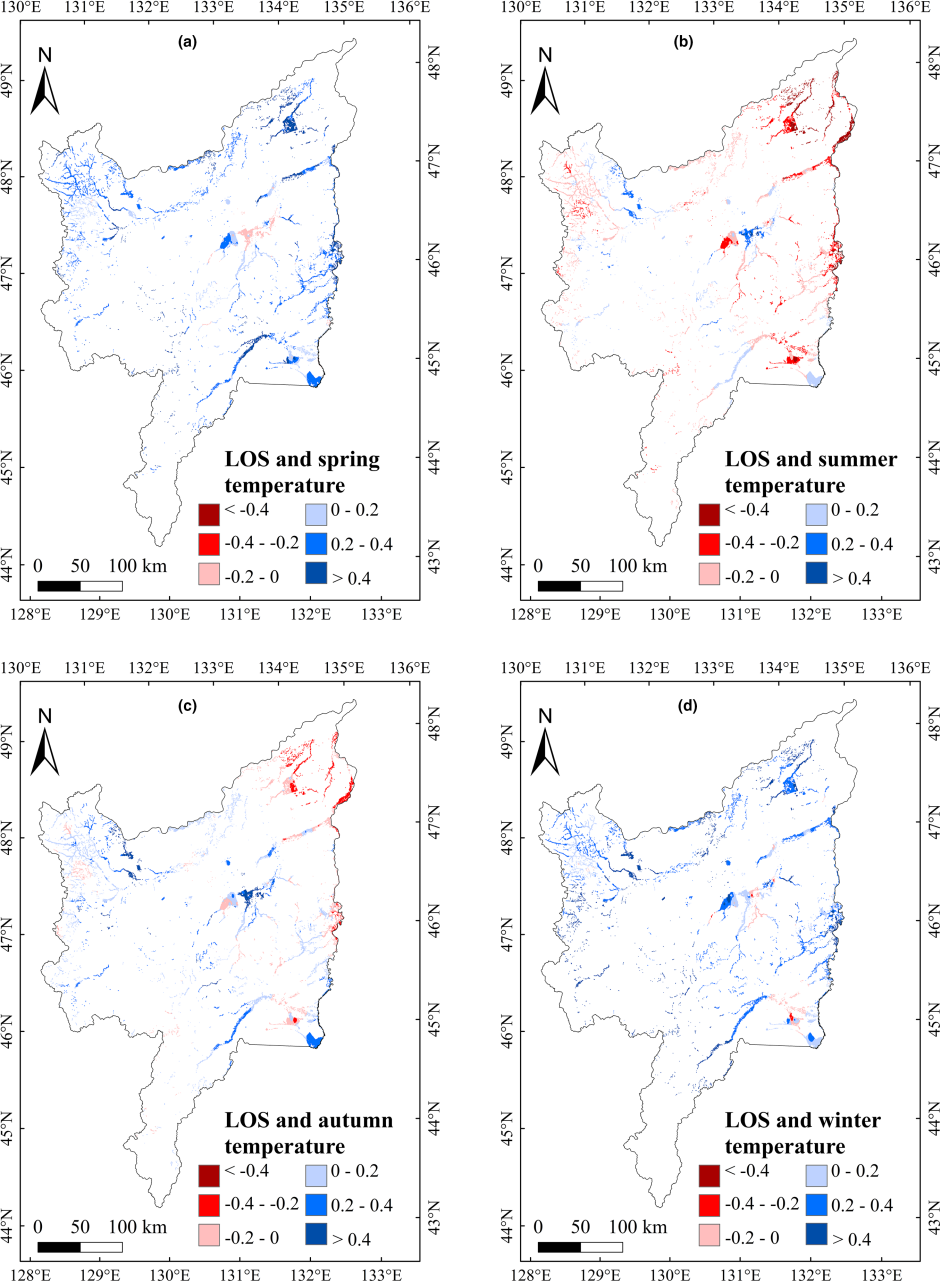
Correlations between LOS and seasonal average temperature in marshes of Sanjiang Plain (2001–2020)

To explore the impacts of climatic variation on vegetation phenology in marshes of Sanjiang Plain, we analyzed the impacts of annual precipitation and temperature on vegetation phenology during 2001–2020. The correlation between annual precipitation and phenology was not significant, but the annual temperature showed significantly (*p* < .05) negative and positive relationship with the SOS and LOS, respectively (Table 1). We further explored the relationship between the temperature and precipitation in different season on marsh vegetation phenology, and the results showed that the marsh SOS had significant (*p* < .05) negative relationships with the temperature in spring and winter, and weak positive relationships with summer and autumn temperatures (Table 1). The EOS had a significant positive correlation with autumn temperature. The marsh LOS was negatively correlated with summer temperature; however, LOS correlations with temperature in other seasons were positive. The LOS correlations between the spring and winter temperature were significant (*p* < .05). For the effects of precipitation, we found no significant relationships between vegetation phenology and precipitation in the different seasons (Table 1). For monthly temperature effects, the relationships of SOS with temperature from December to April were significant (*p* < .05). The EOS was positively correlated with temperature from August to October; furthermore, the correlation between EOS with temperature in October was significant (Table 1). The relationship of LOS with temperature from January to April was significantly (*p* < .05) positive. Similar to the seasonal correlation results, the correlation between vegetation phenology and precipitation for each month was insignificant. Spatially, the region with the largest negative correlations between SOS and temperature in spring and winter was concentrated in the eastern Sanjiang Plain (Figure 4). The region with the largest positive correlation between EOS and temperature in autumn was located east of Sanjiang Plain (Figure 5); furthermore, the region with the largest positive correlations between LOS and temperature in spring and winter was located east of Sanjiang Plain (Figure 6).

**TABLE 1:** Correlation coefficients between vegetation phenology and temperature and precipitation averaged from all the pixels in marshes of Sanjiang Plain, China (2001–2020)

	SOS		EOS		LOS	
	Precipitation	Temperature	Precipitation	Temperature	Precipitation	Temperature
Annual	−0.36	−0.64**	−0.09	0.22	0.22	0.69**
Spring	−0.22	−0.78**	0.06	−0.24	0.22	0.44*
Summer	−0.24	0.06	−0.19	0.28	0.09	−0.12
Autumn	−0.22	0.01	0.09	0.57**	0.24	0.27
Winter	0.34	−0.56*	0.15	0.00	−0.12	0.45*
January	0.09	−0.44*	−0.43	0.15	−0.41	0.47*
February	0.28	−0.60**	0.39	−0.05	0.08	0.44*
March	0.35	−0.81**	0.10	−0.04	−.020	0.61**
April	−0.03	−0.69**	−0.19	−0.15	−0.13	0.45*
May	−0.30	0.09	0.11	−0.42	0.32	−0.42
June	−0.06	0.14	−0.01	0.05	0.24	−0.26
July	−0.08	−0.31	−0.33	0.23	−0.42	0.38
August	−0.33	0.21	−0.04	0.41	0.29	−0.21
September	−0.04	0.10	0.36	0.31	0.39	0.25
October	−0.21	0.07	−0.39	0.44*	−0.08	0.25
November	−0.33	−0.08	0.08	−0.04	0.31	0.07
December	0.22	−0.45*	0.20	−0.11	−0.02	0.27

*Note*: ** and * represent significantly at the levels of *p* < .01 and .05.

## 4 DISCUSSION

### 4.1 The spatiotemporal variations of vegetation phenology in marshes of Sanjiang Plain

Spatial distribution was analyzed in terms of multiyear average vegetation phenology. The region with earlier SOS was mainly distributed in the east of study area (Figure 2a), which was similar to the result of Shen, Liu, et al. (2019). The region with later EOS was concentrated in the east (Figure 2b), and the region with longer LOS was mostly distributed in the east of Sanjiang Plain (Figure 2c). These indicate that the later EOS and earlier SOS may explain longer LOS in the east of study area. This is because that the climate in Sanjiang Plain is a monsoon climate (Jin et al., 2016), and the hydrothermal conditions are better and more suitable for vegetation growth in the east of this region (Liu et al., 2022). Therefore, the SOS of marsh vegetation was early, and the EOS was delayed; thereby, extending the LOS in the east of Sanjiang Plain. Spatially, the region with the largest advancing SOS trend was distributed north of Sanjiang Plain (Figure 2d), confirming the finding of previous research (Shen, Liu, et al., 2019).

### 4.2 Relationship of marsh phenology with climatic factors

The annual temperature showed significantly negative and positive relationship with the SOS and LOS, respectively (Table 1), indicating that the rise in annual temperature can advance the SOS and increase the LOS. The SOS of marsh vegetation was significantly and negatively correlated with temperature during winter and spring, and correlated negligibly with precipitation in each season, indicating that the rise in temperature in spring and winter can advance the SOS. However, precipitation was not the main element influencing SOS in the marsh vegetation of Sanjiang Plain. It may be because that water is relatively abundant in marshes of Sanjiang Plain (Jin et al., 2016; Liu et al., 2022), and thus precipitation has no significant effects on marsh vegetation growth in Sanjiang Plain. By contrast, temperature plays an important role in many developmental biological processes of marsh vegetation (Badeck et al., 2004). The Sanjiang Plain is a temperate and relatively cold region, and the spring leaf onset of marsh plants generally require heat accumulation in this region (Geng et al., 2020). The correlations between SOS and temperature from December to April were significantly (*p* < .05) negative, suggesting that increased temperature from December to April could advance the SOS in marshes of Sanjiang Plain (Shen, Liu, et al., 2019). Our results suggest that increases in spring and winter temperatures may reduce frost and promote heat accumulation to initiate green‐up (Luedeling et al., 2013; Shen, Jiang, Lu, & Zhang, 2022). The EOS had a significantly positive relationship with autumn temperature but had negligible correlation with precipitation, indicating that the rise in autumn temperature may delay the EOS in marshes of Sanjiang Plain. However, this result was different from a previous study (Liu et al., 2016), in which precipitation was the main determinant of grassland EOS and that increasing precipitation can alleviate water stress and delay EOS. The marsh ecosystem has sufficient water compared with the arid grassland ecosystem (Liu et al., 2022; Ma, Shen, et al., 2022; Ma, Xia, et al., 2022; Shen, Liu, Zhang, et al., 2022). Therefore, precipitation in autumn may have no significant impact on the marsh EOS. The increase in autumn temperature can slow down the degradation rate of chlorophyll during leaf senescence and reduce frost (Liu et al., 2016); thereby, delaying the EOS of marsh vegetation. Furthermore, the relationship between temperature in October and EOS was significant (*p* < .05), suggesting that the increased temperature in October could significantly delay the marsh EOS in Sanjiang Plain. The significantly positive correlations between LOS and temperature in spring and winter showed that warming temperature in spring and winter can increase LOS in marshes of Sanjiang Plain. This may be because the temperature rising is conducive to the heat accumulation of vegetation and the reduction of frost, thereby prolonging the LOS of marsh vegetation in this region. The LOS showed significantly positive correlations with temperature from January to April (Table 1), suggesting that increase in temperature from January to April can advance the SOS and could also prolong marsh LOS in study region. Our results showed that climate affects the LOS to a certain extent, specifically by affecting the SOS of marshes in study region.

**FIGURE 7:**
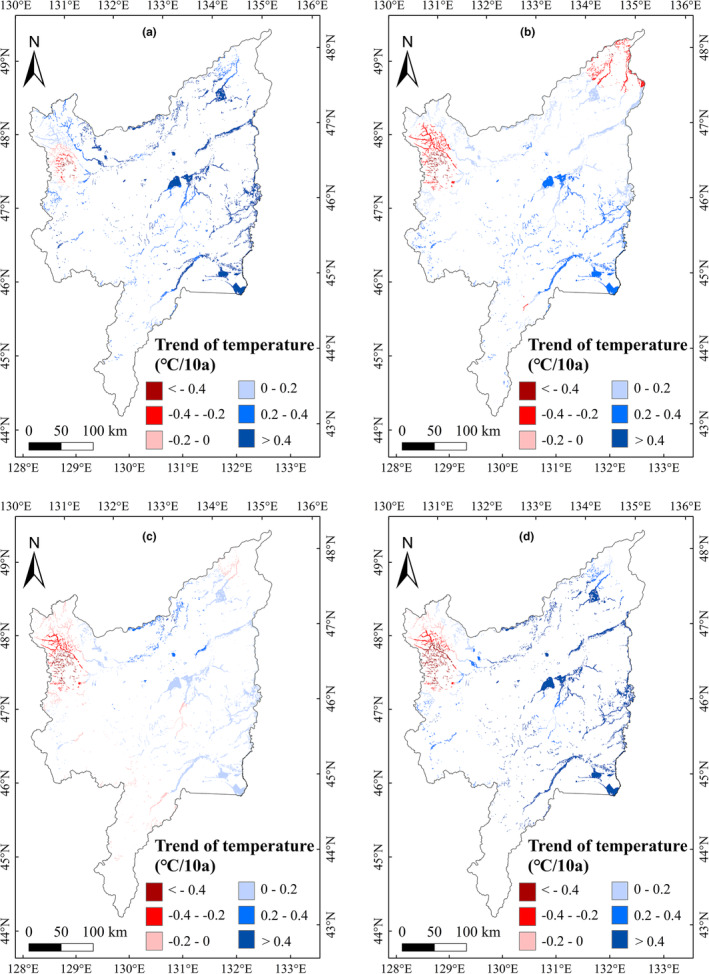
Temperature variation trends in spring (a), summer (b), autumn (c), and winter (d) in marshes of Sanjiang Plain during 2001–2020

In terms of multiyear changes in climatic factors, the average temperature from January to April showed increasing trends and the precipitation in June, August, September, and November showed significant increasing trends (Table 2). Considering that the SOS and LOS had negative–positive correlations with temperature from January to April (Table 1), we concluded that an increase in temperature from January to April could advance SOS and extend LOS of marsh vegetation in Sanjiang Plain. The temperature in July significantly increased from 2001 to 2020 (Table 2), and temperature in July was positively correlated with the EOS (Table 1). Therefore, the increase in average temperature in July could account for the delayed EOS in study region. Spatially, the region with the largest increase in average temperature was located east of Sanjiang Plain in spring, summer, autumn, and winter (Figure 7). Spring and winter temperatures were significantly negatively correlated with marsh SOS (Figure 4), and positively correlated with marsh LOS in the east of study region (Figure 6). Consequently, the increasing spring and winter temperatures may explain the advance of SOS and the increase in LOS in eastern Sanjiang Plain. Similarly, the highest positive correlation between EOS and temperature in autumn was located in eastern Sanjiang Plain (Figure 5c), indicating that the rising temperature in autumn may explain the delay of EOS in the east of Sanjiang Plain.

**TABLE 2:** Temporal trends in climatic factors (temperature (°C/a) and precipitation (mm/a)) in the marsh of Sanjiang Plain during 2001–2020

	Precipitation	Temperature
January	−0.32	0.08
February	−0.10	0.04
March	−0.57	0.10
April	−0.32	0.03
May	2.38	−0.01
June	3.52*	−0.09
July	0.69	0.07*
August	4.76*	−0.00
September	3.82**	0.01
October	−0.04	0.00
November	1.11*	−0.00
December	0.16	−0.01

*Note*: ** and * represent significantly at the levels of *p* < .01 and .05.

### 4.3 Limitations

We should note that current work could have some limitations. First, the marsh distribution and NDVI satellite data used in this study may have some uncertainty due to the effects of some factors such as clouds and solar altitude angle. Second, the phenology results extracted by MOD13Q1 NDVI data might have some uncertainty due to their relatively low temporal–spatial resolution, and future studies are still needed to confirm the phenological results in this study by using or combining other vegetation index data. Third, the effects of human activities on phenological results cannot be completely excluded although this study used the unchanged marsh to reduce the human impacts on the results. In addition, climate data from limited climate stations may impact our results, and future research is still needed to further confirm the research results of this paper by using more climate data. Finally, this study only analyzed the vegetation phenology responses to precipitation and temperature. However, other climatic factors, such as solar radiation, may also affect the vegetation phenology in marshes of Sanjiang Plain. Future researches are needed to analyze the effects of other environmental factors on vegetation phenology in marshes of Sanjiang Plain.

## 5 CONCLUSION

From 2001 to 2020, the long‐term average SOS and EOS were approximately 122 and 296 DOY in Sanjiang Plain, respectively. The LOS of marsh vegetation in study region was approximately 174 days. The SOS advanced by 0.30 days/a, EOS delayed by 0.23 days/a, and LOS increased significantly (*p* < .05) by 0.53 days/a in marshes of Sanjiang Plain. Spatially, the regions with the largest advancing trend of SOS and largest delaying trend of EOS were concentrated in northern Sanjiang Plain. For the effects of climate change, an increase in temperature from December to April may advance the SOS, thereby increasing the LOS. The rise in temperature in October can delay the EOS in marshes of Sanjiang Plain. Spatially, the increase in average temperature in spring and winter may explain the advance of SOS and the increase in LOS in eastern Sanjiang Plain. The temperature rise in autumn may explain the delay of EOS in eastern Sanjiang Plain. This study highlights the importance of seasonal climatic conditions in vegetation phenology model of marshes and indicates that the different climate impacts on the phenology of marsh vegetation in different regions of Sanjiang Plain should be adequately considered to assess the influences of future climate changes on marsh ecosystems in this region.

## AUTHOR CONTRIBUTIONS

**Yiwen Liu:** Conceptualization (equal); data curation (lead); funding acquisition (equal); methodology (equal); writing – original draft (lead). **Xiangjin Shen:** Conceptualization (equal); data curation (equal); funding acquisition (lead); methodology (lead); writing – review and editing (equal). **Jiaqi Zhang:** Conceptualization (equal); data curation (equal); writing – review and editing (equal). **Yanji Wang:** Conceptualization (equal); data curation (equal); writing – review and editing (equal). **Liyuan Wu:** Conceptualization (equal); data curation (equal); writing – review and editing (equal). **Rong Ma:** Conceptualization (equal); data curation (equal); writing – review and editing (equal). **Xianguo Lu:** Supervision (equal). **Ming Jiang:** Supervision (equal).

## CONFLICT OF INTEREST

The authors declare no conflict of interest.

## Data Availability

The climatic data used in this study were downloaded from the China Meteorological Administration (http://www.nmic.cn/en), and the MODIS NDVI data were obtained from the Goddard Space Flight Center (https://ladsweb.modaps.eosdis.nasa.gov/). The marsh distribution data were downloaded from National Earth System Science Data Center (http://www.geodata.cn/index.html).

## References

[ece39755-bib-0002] Badeck, F. W. , Bondeau, A. , Böttcher, K. , Doktor, D. , Lucht, W. , Schaber, J. , & Sitch, S. (2004). Responses of spring phenology to climate change. The New Phytologist, 162, 295–309.

[ece39755-bib-0003] Bhandari, S. , Phinn, S. , & Gill, T. (2011). Assessing viewing and illumination geometry effects on the MODIS vegetation index (MOD13Q1) time series: Implications for monitoring phenology and disturbances in forest communities in Queensland, Australia. International Journal of Remote Sensing, 32, 7513–7538.

[ece39755-bib-0004] Cao, R. , Chen, Y. , Shen, M. , Chen, J. , Zhou, J. , Wang, C. , & Yang, W. (2018). A simple method to improve the quality of NDVI time‐series data by integrating spatiotemporal information with the Savitzky‐Golay filter. Remote Sensing of Environment, 217, 244–257.

[ece39755-bib-0005] Chatterjee, K. , Bandyopadhyay, A. , Ghosh, A. , & Kar, S. (2015). Assessment of environmental factors causing wetland degradation, using Fuzzy Analytic Network Process: A case study on Keoladeo National Park, India. Ecological Modelling, 316, 1–13.

[ece39755-bib-0009] Duan, S. , He, H. S. , & Spetich, M. (2018). Effects of growing‐season drought on phenology and productivity in the west region of central hardwood forests, USA. Forests, 9, 377.

[ece39755-bib-0010] Enkhzaya, T. , & Tateishi, R. (2010). Identification of cultivated area based on Phenological features in arid and semi arid area in Asia. The International Archives of the Photogrammetry, Remote Sensing and Spatial Information Sciences, 38, 473–476.

[ece39755-bib-0011] Erwin, K. L. (2009). Wetlands and global climate change: The role of wetland restoration in a changing world. Wetlands Ecology and Management, 17, 71–84.

[ece39755-bib-0012] Galvão, L. S. , Breunig, F. M. , Teles, T. S. , Gaida, W. , & Balbinot, R. (2016). Investigation of terrain illumination effects on vegetation indices and VI‐derived phenological metrics in subtropical deciduous forests. GIScience & Remote Sensing, 53, 360–381.

[ece39755-bib-0013] Geng, X. , Fu, Y. H. , Hao, F. , Zhou, X. , Zhang, X. , Yin, G. , Vitasse, Y. , Piao, S. , Niu, K. , De Boeck, H. J. , Menzel, A. , & Peñuelas, J. (2020). Climate warming increases spring phenological differences among temperate trees. Global Change Biology, 26, 5979–5987. 32757456 10.1111/gcb.15301

[ece39755-bib-0014] Guo, J. , & Hu, Y. (2022). Spatiotemporal variations in satellite‐derived vegetation Phenological parameters in Northeast China. Remote Sensing‐Basel., 14, 705.

[ece39755-bib-0015] Hao, Q. J. , Wang, Y. S. , Song, C. C. , & Huang, Y. (2006). Contribution of winter fluxes to the annual CH4, CO2 and N2O emissions from freshwater marshes in the Sanjiang Plain. Journal of Environmental Sciences, 18, 270–275.

[ece39755-bib-0016] Hu, K. , Zhang, Z. , Fang, H. , Lu, Y. , Gu, Z. , & Gao, M. (2021). Spatio‐temporal characteristics and driving factors of the foliage clumping index in the Sanjiang Plain from 2001 to 2015. Remote Sensing‐Basel., 13, 2797.

[ece39755-bib-0017] Hu, Z. , Borsje, B. W. , van Belzen, J. , Willemsen, P. W. , Wang, H. , Peng, Y. , Yuan, L. , Dominicis, M. D. , Wolf, J. , Temmerman, S. , & Bouma, T. J. (2021). Mechanistic modeling of marsh seedling establishment provides a positive outlook for coastal wetland restoration under global climate change. Geophysical Research Letters, 48, e2021GL095596.

[ece39755-bib-0018] Hwang, T. , Band, L. E. , Miniat, C. F. , Song, C. , Bolstad, P. V. , Vose, J. M. , & Love, J. P. (2014). Divergent phenological response to hydroclimate variability in forested mountain watersheds. Global Change Biology, 20, 2580–2595. 24677382 10.1111/gcb.12556

[ece39755-bib-0019] Jin, C. , Xiao, X. , Dong, J. , Qin, Y. , & Wang, Z. (2016). Mapping paddy rice distribution using multi‐temporal Landsat imagery in the Sanjiang Plain, Northeast China. Frontiers of Earth Science, 10, 49–62. 27695637 10.1007/s11707-015-0518-3 PMC5042212

[ece39755-bib-0020] Junk, W. J. , An, S. , Finlayson, C. M. , Gopal, B. , Květ, J. , Mitchell, S. A. , Mitsch, W. J. , & Robarts, R. D. (2013). Current state of knowledge regarding the world's wetlands and their future under global climate change: A synthesis. Aquatic Sciences, 75, 151–167.

[ece39755-bib-0021] Lebrini, Y. , Boudhar, A. , Hadria, R. , Lionboui, H. , Elmansouri, L. , Arrach, R. , Ceccato, P. , & Benabdelouahab, T. (2019). Identifying agricultural systems using SVM classification approach based on phenological metrics in a semi‐arid region of Morocco. Earth Systems and Environment, 3, 277–288.

[ece39755-bib-0022] Lewitus, A. J. , Koepfler, E. T. , & Morris, J. T. (1998). Seasonal variation in the regulation of phytoplankton by nitrogen and grazing in a salt‐smarsh estuary. Limnology and Oceanography, 43, 636–646.

[ece39755-bib-0023] Li, Z. , Tang, H. , Yang, P. , Wu, W. , Chen, Z. , Zhou, Q. , Zhang, L. , & Zou, J. (2012). Spatio‐temporal responses of cropland phenophases to climate change in Northeast China. Journal of Geographical Sciences, 22, 29–45.

[ece39755-bib-0024] Liu, Q. , Fu, Y. H. , Zeng, Z. , Huang, M. , Li, X. , & Piao, S. (2016). Temperature, precipitation, and insolation effects on autumn vegetation phenology in temperate China. Global Change Biology, 22, 644–655. 26340580 10.1111/gcb.13081

[ece39755-bib-0025] Liu, Y. , Shen, X. , Wang, Y. , Zhang, J. , Ma, R. , Lu, X. , & Jiang, M. (2022). Spatiotemporal variation in aboveground biomass and its response to climate change in the marsh of Sanjiang Plain. Frontiers in Plant Science, 13, 1973. 10.3389/fpls.2022.920086 PMC9253693 35800612

[ece39755-bib-0026] Luedeling, E. , Guo, L. , Dai, J. , Leslie, C. , & Blanke, M. M. (2013). Differential responses of trees to temperature variation during the chilling and forcing phases. Agricultural and Forest Meteorology, 181, 33–42.

[ece39755-bib-0027] Luo, C. , Fu, X. , Zeng, X. , Cao, H. , Wang, J. , Ni, H. , Qu, Y. , & Liu, Y. (2022). Responses of remnant wetlands in the Sanjiang Plain to farming‐landscape patterns. Ecological Indicators, 135, 108542.

[ece39755-bib-0028] Luo, Y. (2007). Terrestrial carbon–cycle feedback to climate warming. Annual Review of Ecology, Evolution, and Systematics, 38, 683–712.

[ece39755-bib-0029] Ma, R. , Shen, X. , Zhang, J. , Xia, C. , Liu, Y. , Wu, L. , Wang, Y. , Jiang, M. , & Lu, X. (2022). Variation of vegetation autumn phenology and its climatic drivers in temperate grasslands of China. International Journal of Applied Earth Observation and Geoinformation, 114, 103064.

[ece39755-bib-0030] Ma, R. , Xia, C. , Liu, Y. , Wang, Y. , Zhang, J. , Shen, X. , Lu, X. , & Jiang, M. (2022). Spatiotemporal change of net primary productivity and its response to climate change in temperate grasslands of China. Frontiers in Plant Science, 13. 10.3389/fpls.2022.899800 PMC9171389 35685016

[ece39755-bib-0031] Mao, D. , Wang, Z. , Du, B. , Li, L. , Tian, Y. , Jia, M. , Zeng, Y. , Song, K. , Jiang, M. , & Wang, Y. (2020). National wetland mapping in China: A new product resulting from object based and hierarchical classification of Landsat 8 OLI images. ISPRS Journal of Photogrammetry and Remote Sensing, 164, 11–25.

[ece39755-bib-0032] Marani, M. , Da Lio, C. , & D'Alpaos, A. (2013). Vegetation engineers marsh morphology through multiple competing stable states. Proceedings of the National Academy of Sciences of the United States of America, 110, 3259–3263. 23401529 10.1073/pnas.1218327110 PMC3587243

[ece39755-bib-0034] Mo, Y. , Momen, B. , & Kearney, M. S. (2015). Quantifying moderate resolution remote sensing phenology of Louisiana coastal marshes. Ecological Modelling, 312, 191–199.

[ece39755-bib-0035] Muche, M. E. , Hutchinson, S. L. , Hutchinson, J. S. , & Johnston, J. M. (2019). Phenology‐adjusted dynamic curve number for improved hydrologic modeling. Journal of Environmental Management, 235, 403–413. 30708277 10.1016/j.jenvman.2018.12.115 PMC6747703

[ece39755-bib-0036] Natuhara, Y. (2013). Ecosystem services by paddy fields as substitutes of natural wetlands in Japan. Ecological Engineering, 56, 97–106.

[ece39755-bib-0037] Oliveira, T. C. D. , Ferreira, E. , & Dantas, A. A. A. (2016). Temporal variation of normalized difference vegetation index (NDVI) and calculation of the crop coefficient (K c) from NDVI in areas cultivated with irrigated soybean. Ciência Rural, 46, 1683–1688.

[ece39755-bib-0038] Piao, S. , Liu, Z. , Wang, T. , Peng, S. , Ciais, P. , Huang, M. , Ahlstrom, A. , Burkhart, J. F. , Chevallier, F. , Janssens, I. A. , Jeong, S. , Lin, X. , Mao, J. , Miller, J. , Mohammat, A. , Myneni, R. B. , Peñuelas, J. , Shi, X. , Stohl, A. , … Tans, P. P. (2017). Weakening temperature control on the interannual variations of spring carbon uptake across northern lands. Nature Climate Change, 7, 359–363.

[ece39755-bib-0040] Piao, S. L. , Fang, J. Y. , Zhou, L. M. , Ciais, P. , & Zhu, B. (2006). Variations in satellite‐derived phenology in China's temperate vegetation. Global Change Biology, 12, 672–685.

[ece39755-bib-0041] Pringle, M. J. , Denham, R. J. , & Devadas, R. (2012). Identification of cropping activity in central and southern Queensland, Australia, with the aid of MODIS MOD13Q1 imagery. International Journal of Applied Earth Observation and Geoinformation, 19, 276–285.

[ece39755-bib-0042] Saderne, V. , Fusi, M. , Thomson, T. , Dunne, A. , Mahmud, F. , Roth, F. , Carvalho, S. , & Duarte, C. M. (2021). Total alkalinity production in a mangrove ecosystem reveals an overlooked blue carbon component. Limnology and Oceanography Letters, 6, 61–67.

[ece39755-bib-0043] Sarkar, U. K. , & Borah, B. C. (2018). Flood plain wetland fisheries of India: With special reference to impact of climate change. Wetlands Ecology and Management, 26, 1–15.

[ece39755-bib-0044] Sarvia, F. , De Petris, S. , & Borgogno‐Mondino, E. (2021). Exploring climate change effects on vegetation phenology by MOD13Q1 data: The Piemonte region case study in the period 2001–2019. Agronomy, 11, 555.

[ece39755-bib-0045] Shen, M. , Chen, J. , Zhu, X. , & Tang, Y. (2009). Yellow flowers can decrease NDVI and EVI values: Evidence from a field experiment in an alpine meadow. Canadian Journal of Remote Sensing, 35, 99–106.

[ece39755-bib-0047] Shen, X. , Jiang, M. , Lu, X. , Liu, X. , Liu, B. , Zhang, J. , Wang, X. , Tong, S. , Lei, G. , Wang, S. , Tong, C. , Fan, H. , Tian, K. , Wang, X. , Hu, Y. , Xie, Y. , Ma, M. , Zhang, S. , Cao, C. , & Wang, Z. (2021). Aboveground biomass and its spatial distribution pattern of herbaceous marsh vegetation in China. Science China Earth Sciences, 64, 1115–1125.

[ece39755-bib-0048] Shen, X. , Jiang, M. , & Lu, X. (2022). Diverse impacts of day and night temperature on spring phenology in freshwater marshes of the Tibetan plateau. Limnology and Oceanography Letters.

[ece39755-bib-0049] Shen, X. , Liu, B. , Henderson, M. , Wang, L. , Jiang, M. , & Lu, X. (2022). Vegetation greening, extended growing seasons, and temperature feedbacks in warming temperate grasslands of China. Journal of Climate, 35, 5103–5117.

[ece39755-bib-0050] Shen, X. , Liu, B. , Henderson, M. , Wang, L. , Wu, Z. , Wu, H. , Jiang, M. , & Lu, X. (2018). Asymmetric effects of daytime and nighttime warming on spring phenology in the temperate grasslands of China. Agricultural and Forest Meteorology, 259, 240–249.

[ece39755-bib-0051] Shen, X. , Liu, B. , Xue, Z. , Jiang, M. , Lu, X. , & Zhang, Q. (2019). Spatiotemporal variation in vegetation spring phenology and its response to climate change in freshwater marshes of Northeast China. Science of The Total Environment, 666, 1169–1177. 30970482 10.1016/j.scitotenv.2019.02.265

[ece39755-bib-0052] Shen, X. , Liu, Y. , Zhang, J. , Wang, Y. , Ma, R. , Liu, B. , Ln, X. , & Jiang, M. (2022). Asymmetric impacts of diurnal warming on vegetation carbon sequestration of marshes in the Qinghai Tibet plateau. Global Biogeochemical Cycles, 36, e2022GB007396.

[ece39755-bib-0053] Shen, X. , Xue, Z. , Jiang, M. , & Lu, X. (2019). Spatiotemporal change of vegetation coverage and its relationship with climate change in freshwater marshes of Northeast China. Wetlands, 39, 429–439. 10.1016/j.scitotenv.2019.02.265 30970482

[ece39755-bib-0054] Shen, X. J. , Liu, B. H. , Jiang, M. , Wang, Y. J. , Wang, L. , Zhang, J. Q. , & Lu, X. G. (2021). Spatiotemporal change of marsh vegetation and its response to climate change in China from 2000 to 2019. Journal of Geophysical Research – Biogeosciences, 126, e2020JG006154.

[ece39755-bib-0055] Smith, A. J. , & Kirwan, M. L. (2021). Sea level‐driven marsh migration results in rapid net loss of carbon. Geophysical Research Letters, 48, e2021GL092420.

[ece39755-bib-0056] Song, K. , Wang, Z. , Du, J. , Liu, L. , Zeng, L. , & Ren, C. (2014). Wetland degradation: Its driving forces and environmental impacts in the Sanjiang Plain, China. Environmental Management, 54, 255–271. 24844462 10.1007/s00267-014-0278-y

[ece39755-bib-0057] Su, M. , Huang, X. , Xu, Z. , Zhu, W. , & Lin, Z. (2022). A decrease in the daily maximum temperature during global warming hiatus causes a delay in spring phenology in the China–DPRK–Russia cross‐border area. Remote Sensing‐Basel., 14, 1462.

[ece39755-bib-0058] Sun, L. , & Song, C. (2008). Evapotranspiration from a freshwater marsh in the Sanjiang Plain, Northeast China. Journal of Hydrology, 352, 202–210.

[ece39755-bib-0059] Vázquez‐Lule, A. , & Vargas, R. (2021). Biophysical drivers of net ecosystem and methane exchange across phenological phases in a tidal salt marsh. Agricultural and Forest Meteorology, 300, 108309.

[ece39755-bib-0061] Wang, Z. , Song, K. , Ma, W. , Ren, C. , Zhang, B. , Liu, D. , Chen, J. , & Song, C. (2011). Loss and fragmentation of marshes in the Sanjiang Plain, Northeast China, 1954–2005. Wetlands, 31, 945–954.

[ece39755-bib-0063] Wu, Y. , Xu, N. , Wang, H. , Li, J. , Zhong, H. , Dong, H. , Zeng, Z. , & Zong, C. (2021). Variations in the diversity of the soil microbial community and structure under various categories of degraded wetland in Sanjiang Plain, northeastern China. Land Degradation and Development, 32, 2143–2156.

[ece39755-bib-0065] Zeng, J. , Sun, Y. , Cao, P. , & Wang, H. (2022). A phenology‐based vegetation index classification (PVC) algorithm for coastal salt marshes using Landsat 8 images. International Journal of Applied Earth Observation and Geoinformation, 110, 102776.

